# Cost-effectiveness of physical activity interventions for prevention and management of cognitive decline and dementia—a systematic review

**DOI:** 10.1186/s13195-023-01286-7

**Published:** 2023-09-25

**Authors:** Weixin Li, Kun-Woo Rafael Kim, Donglan Zhang, Bian Liu, Christine M. Dengler-Crish, Ming Wen, Lu Shi, Xi Pan, Yian Gu, Yan Li

**Affiliations:** 1 Department of Population Health Science and Policy, Icahn School of Medicine at Mount Sinai, New York, NY USA; 2 The Graduate School of Biomedical Sciences, Icahn School of Medicine at Mount Sinai, New York, NY USA; 3 Department of Health Policy, Vanderbilt University School of Medicine, Nashville, TN USA; 4 Division of Health Services Research, Department of Foundations of Medicine, New York University Long Island School of Medicine, Mineola, NY USA; 5 Department of Pharmaceutical Sciences, Northeast Ohio Medical University, Rootstown, OH USA; 6 Department of Sociology, The University of Hong Kong, Hong Kong, China; 7 Department of Sociology, University of Utah, Salt Lake City, UT USA; 8 Department of Public Health Sciences, Clemson University, Clemson, SC USA; 9 Department of Sociology, Texas State University, San Marcos, TX USA; 10 Department of Neurology, Department of Epidemiology, Columbia University, New York, NY USA; 11 Department of Health Policy and Management, School of Public Health, Shanghai Jiao Tong University School of Medicine, 227 S. Chongqing Rd, Shanghai, China

**Keywords:** Exercise, Cognitive health, Dementia, Aging, Cost-effectiveness

## Abstract

**Background:**

Although increasing physical activity (PA) has been suggested to prevent and manage cognitive decline and dementia, its economic impact on healthcare systems and society is largely unknown. This study aimed to summarize evidence on the cost-effectiveness of PA interventions to prevent and manage cognitive decline and dementia.

**Methods:**

Electronic databases, including PubMed/MEDLINE, Embase, and ScienceDirect, were searched from January 2000 to July 2023. The search strategy was driven by a combination of subject-heading terms related to physical activity, cognitive function, dementia, and cost-effectiveness. Selected studies were included in narrative synthesis, and extracted data were presented in narrative and tabular forms. The risk of bias in each study was assessed using the Consolidated Health Economic Evaluation Reporting Standards and Consensus on Health Economic Criteria list.

**Results:**

Five of the 11 identified studies focused on individuals with existing dementia. Six of the 11 identified studies focused on individuals with no existing dementia, including 3 on those with mild cognitive impairment (MCI), and 3 on those with no existing MCI or dementia. PA interventions focused on individuals with no existing dementia were found to be cost-effective compared to the control group. Findings were mixed for PA interventions implemented in individuals with existing dementia.

**Conclusions:**

PA interventions implemented before or during the early stage of cognitive impairment may be cost-effective in reducing the burden of dementia. More research is needed to investigate the cost-effectiveness of PA interventions in managing dementia. Most existing studies used short-term outcomes in evaluating the cost-effectiveness of PA interventions in the prevention and management of dementia; future research should consider adding long-term outcomes to strengthen the study design.

**Supplementary Information:**

The online version contains supplementary material available at 10.1186/s13195-023-01286-7.

## Introduction

As the global population ages, the prevalence of dementia continues to rise, which becomes one of the greatest clinical, public health, and social challenges. Worldwide, it is estimated that around 55 million people were living with dementia in 2019, and the number may increase to 139 million in 2050 [1]. Dementia severely erodes functioning and quality of life for people affected and creates devastating burdens and stress for their families and healthcare systems. The economic consequences of dementia are enormous—the global societal cost of dementia in 2019 was estimated to be $1.3 trillion [1]. This brings about a critical question: given the limited healthcare and public health resources, how can we better allocate resources to curb the burden of dementia effectively and efficiently? [2].

Physical activity (PA) is defined as any bodily movement produced by skeletal muscles that results in energy expenditure [3]. Several systematic reviews and meta-analyses suggested that PA may reduce or delay the development of several modifiable risk factors for cognitive declines, such as obesity, diabetes, and hypertension [4–6]. Other literature reviews and studies found that PA interventions could be effective in improving cognition among individuals with mild cognitive impairment (MCI) [7–9] and individuals with dementia [10–12]. Although increasing PA has been proposed to facilitate healthy aging and suggested as a protective factor for cognitive decline and dementia, the economic implications of using PA interventions for reserving cognitive function or reducing the burden of dementia remain unclear. To inform decision-makers on resource allocation, it is necessary to consider whether the effectiveness (or benefit) of PA intervention outweighs its cost, given the preference of the population [13]. In this study, we conduct a systematic review of cost-effectiveness analysis (CEA) of PA interventions for the prevention and management of cognitive decline and dementia. Our objective is to synthesize current evidence on the cost-effectiveness of PA interventions related to cognitive function, MCI, and dementia. We aim to inform future intervention design and policymaking for reducing the burden of dementia. Our systematic review will also shed light on future research directions in the economic evaluation of PA interventions for reducing cognitive decline and preventing and managing dementia.

## Methods

We followed the systematic review’s Preferred Reporting Items for Systematic Reviews and Meta-Analyses (PRISMA) Checklist (Supplemental material) [14]. The review protocol was registered in the PROSPERO system (CRD42022365200).

### Data sources and search strategy

A literature search was conducted using PubMed/MEDLINE, Embase, and ScienceDirect, with publication dates ranging from January 2000 to July 2023. As guidelines for diagnosing and treating MCI and dementia have been evolving, we only included studies over the past two decades and excluded studies before 2000 so that findings from the included studies are relatively comparable [15, 16]. The search strategy was driven by a combination of subject-heading terms related to PA, cognitive function, MCI, dementia, and cost-effectiveness. The following search terms were used in PubMed: ((physical exercise) OR (physical activity) OR (leisure time) OR (sport) OR (muscle stretching exercise) OR (fitness) OR (physical activities) OR (exercise training) OR (physical training)) AND ((quality-adjusted) OR (cost-utility) OR (cost-effectiveness) OR (health economics) OR (economic evaluation)) AND ((mild cognitive impairment) OR (dementia) OR (Alzheimer’s disease)). This approach was adapted accordingly to search on Embase and ScienceDirect and the completed search strategy was listed in Supplementary Table S1. We also searched the reference lists of the selected articles and other review articles to identify studies missing from the database search. The literature pool was exported to EndNote X9.

### Study selection

The inclusion criteria of our population of interest included adults aged over 18 years old. We excluded studies that focus on children and animal models. Interventions of interest included a wide range of PA interventions aimed at preventing dementia or managing existing MCI or dementia. PA interventions included both exercise (i.e., a subset of PA planned and structured to improve or maintain physical fitness) and daily activities (e.g., occupational, sports, household, or other activities that result in energy expenditure) [3]. For comparison, we included adults who received standard-of-care (i.e., standard information or treatments provided by neurologists or clinicians) or different frequency, duration, and intensity of PA interventions. All economic outcomes related to cost-effectiveness were included in the review. Cost-effectiveness was assessed using the incremental cost-effectiveness ratio (ICER), which measures the additional cost required to gain an additional unit of effectiveness or benefit. The ICER was calculated by comparing the costs and outcomes of the intervention with those of the comparator or standard of care [17]. An intervention was considered cost-effective if the calculated ICER was lower than the willingness to pay threshold (i.e., the maximum amount society is willing to pay for an additional unit of health benefit) [17]. Willingness to pay thresholds are not uniformly defined but are often related to the economic wealth of a society or country [17]. We included studies that reported cost-effectiveness given a variety of health outcomes, such as quality-adjusted life years and other relevant outcomes reported in the included studies. Review articles, editorials, letters, research notes, conference abstracts, and protocol-only articles were excluded. Articles not written in English were excluded. Two co-authors (W.L. and K.R.K.) conducted a comprehensive search to identify relevant studies and removed duplicates using the automatic function in EndNote and manual hand search. During the initial screening phase, W.L. and K.R.K. independently assessed titles and abstracts to determine whether studies met the eligibility criteria. During the following screening phase, W.L. and K.R.K. independently examined the full text of the remaining studies to determine eligibility. Disagreements were resolved by consensus or in consultation with a third reviewer (Y.L.). All decisions were recorded through EndNote and Excel spreadsheets.

### Data extraction

Two co-authors (W.L. and K.R.K.) independently extracted data from the selected articles using a standardized data extraction form and recorded results in Microsoft Excel. Discrepancies were resolved through discussion and consensus between the two co-authors or in consultation with a third reviewer (Y.L.). Extracted data included the following: (1) study identification (first author, year, title, country); (2) type of the CEA study, including trial-based CEA (performed alongside clinical trial), model-based CEA (developed using best available evidence from the literature), and “Hybrid” CEA (“In-trial” results extrapolated using modeling techniques) [18]; (3) other study design including time horizon (i.e., the time over which the costs and effects were measured), intervention, sample size, and inclusion criteria; (4) health economic properties (perspective, discount rate, sensitivity analysis, sources of cost data, measures of health outcome); (5) main health economic outcomes (e.g., ICER); and (6) conclusions. All the co-authors checked the extracted data and confirmed the decision.

### Risk of bias assessment

As recommended by the Cochrane collaboration, two co-authors (W.L. and K.R.K.) independently assessed the risk of bias of the included studies using the Consolidated Health Economic Evaluation Reporting Standards (CHEERS) Checklist and Consensus on Health Economic Criteria (CHEC)-list [19]. The CHEER Checklist is a checklist with 24 items designed to focus on the reporting quality of economic evaluations [20]. A study was considered to have good reporting quality if it reported 20–24 items, moderate quality if it reported 14–19 items, and low quality if it reported less than 14 items. The CHEC-list is a checklist with 19 items designed to focus on the methodological quality of economic evaluations [21]. A study was considered to have good methodological quality if it reported 15–19 items, moderate methodological quality if it reported 11–14 items, and low methodological quality if it reported less than 11 items. Disagreements were resolved by consensus or consultation with a third reviewer (Y.L.).

### Equity, diversity, and inclusion statement

Our research team consisted of seven women and three men from a variety of disciplines, including two junior researchers. Our population of interest included all ages, genders, and race/ethnicities.

## Results

**Fig. 1:**
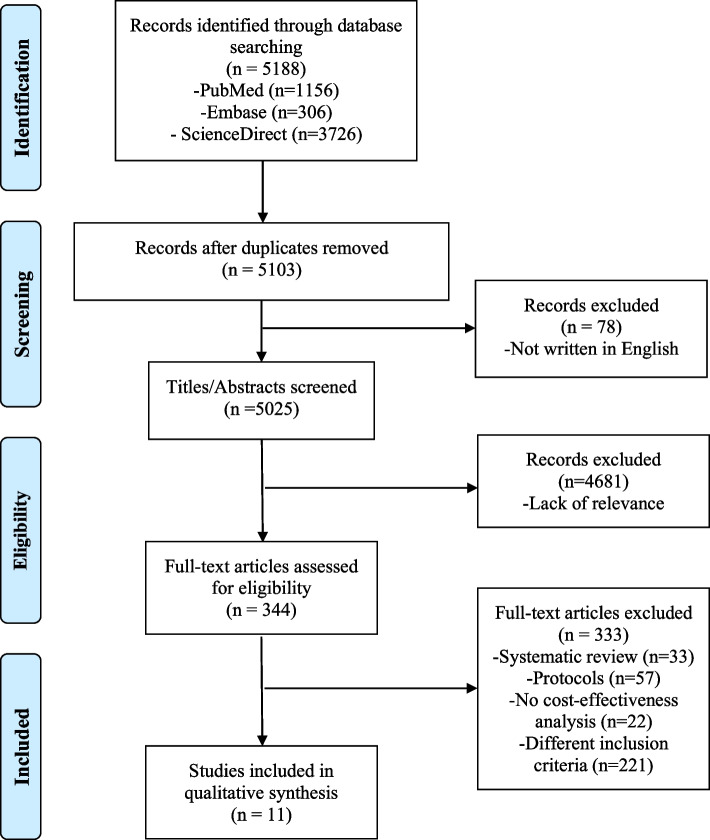
PRISMA flowchart. PRISMA indicates for Preferred Reporting Items for Systematic Reviews and Meta-Analyses

Figure 1 shows the selection flow chart of the studies included in the current review. The literature search identified a total of 5188 studies. After removing duplicates and articles not written in English, two co-authors (W.L. and K.R.K.) independently screened titles and abstracts of the remaining 5103 studies and removed 4681 studies that were not relevant to the topic. Then, two co-authors (W.L. and K.R.K.) independently conducted full-text reviews for the remaining 344 studies and excluded 33 systematics reviews, 57 protocols, 22 studies with no cost-effectiveness analysis, and 221 studies that did not meet the inclusion criteria. In the 23 years, only 11 studies were identified and included in the analysis [22–32].

### Study design and reporting quality

Table 1 summarizes the study design and reporting quality of the 11 identified studies. 8 studies were conducted in a European setting, 2 studies were conducted in Canada, and 1 study was conducted in Japan. 8 studies were trial-based CEA, 2 were model-based CEA, and 1 was a “hybrid” CEA. Among the trial-based CEA, the sample size ranged from 52 to 494 individuals [23–28, 31, 32]. All trial-based CEA investigated the short-term effect ranging from 16 weeks to 12 months. The long-term effect was studied in the model-based and hybrid CEA. One model-based CEA simulated 1000 individuals for 10 years, and the other simulated 1000 individuals for a lifetime [22, 29]. The “hybrid” CEA extended the results of a 2-year clinical trial and generated projections of lifetime outcomes for a simulated cohort of 1,000,000 individuals [30]. All studies had good reporting quality as they reported 20 or more CHEERS Checklist items. The limitations of study reporting quality included not identifying the study as an economic evaluation in the title (*n*= 2) [24, 29], not reporting study perspective (*n*= 2) [24, 29], no justification for not discounting costs or outcomes (*n*= 2) [23, 24], not addressing uncertainties (*n*= 1) [24], no descriptions on assumptions in the analytic solution (*n*= 1) [27], incomplete information on costs (i.e., unit costs and price date) (*n*= 1) [23], and no funding source information (*n*= 1) [32]. All studies had good methodological quality as they scored 15–19 using the CHEC-list. The limitations of study methodology quality included not clearly describing competing alternatives [22, 29], did not choose the most appropriate perspective [24, 29], costs were not measured appropriately in physical units [23, 25], did not perform an incremental analysis of costs and outcomes of alternatives [24], not discounting future costs and outcomes [23, 24], did not conduct sensitivity analysis [24], and not including discussion of generalizability of the results [26].

**Table 1:** Study designs of the identified studies

Study	Country	Design	Time horizon	Sample size	Intervention	Inclusion criteria	CHEERS items	CHEC-list
D’Amico et al. [25] (2016)	UK	Trial-based CEA	3 months	Total: 52 Study group: 30 Control group: 22	Study group: 12-week individually tailored walking program lasting for 20–30 min daily, designed to become progressively more intensive + standard-of-care Control group: standard-of-care	Diagnosis of dementia defined by ICD-10 DCR, or $\ge$ 1 BPSD symptoms, and has a carer willing to participate with the exercise regimen	24	18
Davis et al. [26] (2013)	Canada	Trial-based CEA	6 months	Total: 86 Study groups: 28 in RT group and 30 in AT group Control group: 28	RT group: 2 × 1 h/week resistance training for 6 months AT group: 2 × 1 h/week aerobic training for 6 months Control group: 2 × 1 h/week balance and tone classes for 6 months	Community dwelling women aged 70–80 years, MMSE $\ge$ 24, answered “yes” to the question “Do you have any difficulty with your memory”, scored $\ge$ 6/8 on the Lawton and Brody Instrumental Activities of Daily Living	24	18
Davis et al. [27] (2017)	Canada	Trial-based CEA	12 months	Total: 70 Study group: 25 Control group: 35	Study group: 3 × 1 h/week aerobic training + standard-of-care + education for 6 months Control group: standard-of-care + education	Diagnosis of mild subcortical vascular cognitive impairment: community dwelling older adults with the presence of cognitive syndrome and small vessel ischemic disease or MoCA score < 26, MMSE score $\ge$ 20	23	19
Eckert et al. [28] (2021)	Germany	Trial-based CEA	24 weeks	Total: 118 Study group: 63 Control group: 55	Study group: 12-week home-based exercise (walking, balance, strength training) by sports scientists with home visits and phone calls Control group: unspecific flexibility training	Cognitively impaired geriatric patients discharged from ward rehabilitation, aged $\ge$ 65 years, MMSE score range 17–26, living in the community or in assisted living	24	19
Kato et al [22] (2022)	Japan	Model-based CEA	10 years	Total: simulated 1000	Study group: 90-min weekly session focused on physical and cognitive activities, which was conducted 40 times in the first year. Then, individuals stayed in well or MCI state receive the program twice a year Control group: no intervention	Community-dwelling healthy adults aged 65 years old	24	18
Khan et al. [31] (2019)	UK	Trial-based CEA	12 months	Total: 494 Study group: 329 Control group: 161	Study group: 2 × (1–1.5) h/week supervised exercise for 4 months + 3 × 50 min/week unsupervised exercise for 8 months; combining aerobic and resistance training + standard-of-care Control group: standard-of-care	Mild-to-moderate dementia: diagnosis of dementia defined by DSMIV and standardized MMSE score of > 10	24	19
Pitkälä et al. [24] (2013)	Finland	Trial-based CEA	12 months	Total: 210 Study groups: 70 in GE group and 70 in HE group Control group: 70	GE group: 2 × 1 h/week group-based exercise for 12 months, groups of 10 patients, supervised by 2 physiotherapists and registered nurses HE group: 2 × 1 h/week tailored home-based exercise, supervised by a physiotherapist Control group: standard-of-care	Diagnosis of AD, aged $\ge$ 65 years, has a spouse living together, and the ability to walk independently	20	15
Sopina et al. [32] (2017)	Denmark	Trial-based CEA	16 weeks	Total: 200 Study group: 107 Control group: 93	Study group: 3 × 1 h/week for 16 weeks moderate-to-high intensity aerobic exercises (on bicycle, cross trainer and treadmill supervised by physiotherapist) Control group: standard-of-care	Diagnosis of mild AD, aged 50–90 years, MMSE $\ge$ 20	23	19
Van Santen et al. [23] (2021)	Netherland	Trial-based CEA	6 months	Total: 112 Study group: 73 Control group: 39	Study group: 6-month 2 times/week exergaming (interacting cycling) + regular activity program (music listening, singing, arts & crafts, cooking, gymnastics, and outdoor walking) Control group: regular activity program	Diagnosis of any type of dementia, all ages, community-dwelling, > 2 visit/week at the day-care centers, has an informal caregiver willing to participate	22	17
Baal et al. [29] (2016)	UK	Model-based CEA	Lifetime	Total: simulated 1000	Study group: hypothetical increase of physical activity level by 1 level (out of total 5 levels) Control group: no increase	Simulated 2012 English population aged 40–65 years old	22	17
Wimo et al. [30] (2022)	Sweden	“Hybrid” CEA	Lifetime	Simulated 100,000	Study group: A 2-year multidomain lifestyle intervention (individual and group sessions to foster tailored dietary changes, 1 to 3 aerobic exercise and 2 to 5 resistance training sessions per week, and group and individual cognitive training) Control group: standard-of-care with regular health advice	Adults aged 60 to 77 years that are at risk of dementia (with CAIDE dementia risk scores of at least 6 points and cognition near or slightly below that expected for age)	24	19

Studies were grouped by the study design and then ordered alphabetically within each group. Time horizon indicates the time over which the costs and effects are measured. CHEERS indicates for Consolidated Health Economic Evaluation Reporting Standards; *CHEC-list* Consensus on Health Economic Criteria list; *UK* United Kingdom, *CEA* cost-effectiveness analysis, *MCI* mild cognitive impairment, *ICD-10 DCR* International Statistical Classification of Diseases and Related Health Problems, 10th revision, Diagnostic Criteria for Research (1992), *BPSD* behavioral and psychological symptoms of dementia, *RT* resistance training, *AT* aerobic training, *HE* home-based exercise, *GE* group-based exercise, *MoCA* Montreal Cognitive Assessment, *MMSE* Mini Mental State Examination score, *AD* Alzheimer’s Disease, *DSMIV* Diagnostic and Statistical Manual, 4th Edition, The CAIDE risk score provides an estimate of dementia risk based on several risk factors (age, sex, education, systolic blood pressure, total serum cholesterol, obesity, physical inactivity)

### Population characteristics

Among the 11 studies, 5 studies evaluated the cost-effectiveness of PA interventions as management strategies for patients already diagnosed with dementia, while the remaining studies evaluated PA interventions as prevention strategies in individuals with either MCI (*n* = 3 studies) or no dementia/MCI diagnosis (*n* = 3 studies).

Each study incorporated different inclusion criteria for participant recruitment. Examples of this include the studies by Sopina et al. and Pitkälä et al., which only included patients diagnosed with Alzheimer’s disease (AD) as their sample with current dementia; [24, 32] and the Davis et al. study only considered individuals diagnosed with mild subcortical vascular cognitive impairment as their criteria for a sample with MCI [27]. The Baal et al. study included the whole population of England as a sample with no dementia [29]. Wimo et al. targeted older adults at risk for dementia as determined by a risk score based on age, sex, education, blood pressure, serum cholesterol, obesity, and physical activity as predictors of increased dementia likelihood [30].

### Health economic properties

Table 2 summarizes the identified studies’ health economic properties and cost-effectiveness outcomes. The selected studies were categorized as representing the healthcare sector perspective (i.e., include formal medical costs borne by third-party payers or paid for out-of-pocket by patients) or societal perspective (i.e., also include time costs and effects on future productivity as well as relevant non–health-related impacts in other sectors) [33]. Three studies were conducted from a healthcare sector perspective [26, 27, 32], 3 studies were conducted from a societal perspective [23, 28, 30], and 3 studies used both healthcare sector perspective and societal perspectives [22, 25, 31]. Discounting reflects the loss in economic value that occurs when there is a delay in realizing a benefit or incurring a cost [34]. Five studies reported that discounting was not needed due to the short time horizon used for the analysis, and 4 studies reported specific discount rates. Ten out of the 11 studies also reported sensitivity analyses to address uncertainties (i.e., changes in the results given changes in the input values). The most frequently used measure of health outcome was *quality-adjusted life year* (*n* = 8), which was measured by health-related quality of life using European Quality of Life 5 Dimensions 3 Level Version (*n* = 6), Dementia Quality of Life instrument-proxy (*n* = 1), or both European Quality of Life 5 Dimensions 5 Level Version and European Quality of Life visual analog scales (*n* = 1). Other measures of health outcomes included *behavioral and psychological symptoms of dementia* measured by the Neuropsychiatric Inventory (*n* = 1), executive cognitive function measured by the Stroop Test (*n* = 1), *physical performance* measured by short physical performance battery only (*n* = 2) or with the functional independence measure (*n* = 1), life years (*n* = 1), and Alzheimer’s Disease Assessment Scale (*n* = 1).

**Table 2:** Health economic properties and analytical details of the identified studies

Study	Perspective	Discount rate	Sensitivity analysis	Data source of cost	Measures of health outcome	Main economic outcome and result
D’Amico et al. [25] (2016)	Healthcare sector perspective, societal perspective	3.5%	PSA	Unit cost: Personal Social Services Research Unit compendium for 2011, British National Formulary database Service used: CSRI completed by carer	A clinically significant change in BPSD symptoms (reduction of 3 or more points of NPI); health-related quality of life (measured by DEMQOL-Proxy)	From healthcare payer perspective, study group was dominant for BPSD and QALY From societal perspective, ICERs: CAD$ 421 per significant change of; CAD$ 286440 per QALY WTP threshold: CAD$ 20000/QALY
Davis et al. [26] (2013)	Healthcare sector perspective	Not applied	PSA	Unit cost: British Columbia Medical Services Plan 2013 price list Service used: patient self-complete questionnaires, caregiver survey questionnaire	Changes in executive cognitive function (seconds gained or lost of Stroop Test)	Incremental Stroop Interference time: AT group vs. BAT group was 7.5 s RT group vs. BAT group was 7.8 s The mean total healthcare costs were lower in the AT and RT groups compared with BAT group Study groups (AT and RT) were dominant
Davis et al. [27] (2017)	Healthcare sector perspective	Not applied	PSA	Unit cost: British Columbia Medical Services Plan 2013 price list Service used: telephone interview, patient’s monthly diary of services used, health resource usage questionnaire	Health-related quality of life measured by EQ-5D-3L	ICER: CAD$ 3761 per patient-rated QALY CAD$ 3715 per caregiver-rated QALY WTP threshold: CAD$ 20000/QALY
Eckert et al. [28] (2021)	Societal perspective	Not applied	PSA	Unit cost: Standardized unit cost for German healthcare system (German Federal Statistical Office) Service used: questionnaire for medical and nonmedical services answered by patients or caregivers	A clinically significant change in physical performance (1 point of increase on the SPPB total score); health-related quality of life measured by EQ-5D-3L	The probability of cost-effectiveness referring to physical performance (measured by SPPB score) was 92%, given a decision maker’s WTP threshold of EUR€ 500 per one-point gain on the SPPB score. The probability of cost-effectiveness referring to QALYs was 85% at a WTP threshold of EUR€ 5000 per QALY, and leveled off at 90%, given WTP above EUR€ 20000
Kato et al. [22] (2022)	Healthcare sector perspective, societal perspective	2%	DSA PSA	Unit cost and service used: published literatures	Health-related quality of life measured by EQ-5D-3L	ICER: − 5,740,083 Japanese yen/QALY WTP threshold: 5,000,000 Japanese yen/QALY
Khan et al. [31] (2019)	Healthcare sector perspective, societal perspective	Not applied	DSA PSA	Unit cost: Health and Social Care Information Centre drug costs, NHS Reference Costs trusts schedules, The NHS Hospital and Community Health Services Pay and Prices Index Service used: CSRI	Cognitive outcomes (participant reported ADAS-Cog score); health-related quality of life that measured by EQ-5D-3L	ADAS-Cog score had worsened slightly to 25.2 (standard deviation [SD] 12.3) in the exercise arm and 23.8 (SD 10.4) in the standard-of-care The probability that the exercise program is cost-effective was < 1% across WTP thresholds. incremental net monetary benefit ranged between US$3719 and US$3086 at cost-effectiveness thresholds between US$21450 and US$42900 per QALY
Pitkälä et al. [24] (2013)	NA	NA	NA	Unit cost: Finnish national cost registered 2006 Service used: medical records	Physical functioning (evaluated with the FIM change) and mobility (assessed with the SPPB score)	FIM change (*p* < 0.01): HE group: − 7.1 (95% CI: − 3.7, − 10.5; *p* = 0.004) GE group: − 10.3(95% CI: − 6.7, − 13.9; *p* = 0.12) CG group: − 14.4(95% CI: − 10.9, − 18.0) Costs: HE group: US$25 112 (95% CI: US$17 642 to US$32 581; *p* = .13 vs. CG) GE group: US$22 066 in the GE group (95% CI: US$15 931 to US$28 199; *p* = .03 vs. CG) CG group: US$34 121 (95% CI: US$24 559 to US$43 681)
Sopina et al. [32] (2017)	Healthcare sector perspective	Not applied	PSA	Unit cost and service used: recorded by physiotherapist	Health-related quality of life that EQ-5D-5L and EQ-VAS	The intervention cost was estimated at EUR€608 and EUR€496 per participant, with and without transport cost, respectively. Participants and caregivers in the intervention group reported a small, positive non-significant improvement in EQ-5D-5L and EQ-VAS after 16 weeks. The ICER was estimated at EUR€72 000/quality-adjusted life year using participant-reported outcomes and EUR€87000 using caregiver-reported outcomes
Van Santen et al. [23] (2021)	Societal perspective	NA	PSA	Unit cost: standard prices from the Dutch guidelines for economic evaluations Service used: cost diaries filled out by participants	Health-related quality of life that measured by EQ-5D-3L; physical activity (in minutes) and mobility (based on SPPB score)	ICER: $$-$$ EUR€781/QALY (societal costs were higher and effects were smaller in the exergaming group); EUR€0.70 per one minute gained in physical activity; EUR€533 per one point gained on the and mobility (based on SPPB score)
Baal et al. [29] (2016)	NA	3.5%	DSA, PSA	Unit cost and service used: published literatures	Life years	Incremental Life Years: 0.23 life years Incremental Cost: -£400
Wimo et al. [30] (2022)	Societal perspective	3%	DSA	Unit cost and service used: published literatures	Health-related quality of life that measured by EQ-5D-3L	The FINGER program resulting in savings of 16,928 SEK (2023 US$) and 0.043 QALY gains per person, supporting extended dominance for the FINGER program

Studies were grouped by the study design and then ordered alphabetically within each group (i.e., aligned with Table 1). Dominant means the study group was less costly and more effective than the control group. Not applied means the authors reported that discounting was not appropriate due to the short time horizon used for the analysis. NA means the information is not available in the study. Stroop test is a test of selective attention and conflict resolution

*PSA* probabilistic sensitivity analysis, *DSA* deterministic sensitivity analysis, *ICER* incremental cost-effectiveness ratio, *QALY* quality-adjusted life-year, *EQ-5D-3L* European Quality of Life 5 Dimensions 3 Level Version, *EQ-5D-5L* European Quality of Life 5 Dimensions 5 Level Version, *EQ-VAS* European Quality of Life visual analog scales, *NPI* Neuropsychiatric Inventory, *WTP* willingness-to-pay, *DEMQOL-proxy* Dementia Quality of Life Instrument-proxy, *CSRI* Client Service Receipt Inventory, *BPSD* behavioral and psychological symptoms of dementia, *RT* resistance training, *AT* aerobic training, *BAT* balance and tone classes, *HE* home-based exercise, *GE* group-based exercise, *SIVCI* subcortical ischemic vascular cognitive impairment, *NHS* UK National Health Service, *FIM* Functional Independence Measure, *SPPB* Short Physical Performance Battery, *ADAS-Cog* Alzheimer’s Disease Assessment Scale–Cognitive Subscale, *FINGER* Finnish Geriatric Intervention Study to Prevent Cognitive Impairment and Disability program

### Cost-effectiveness results

Table 3 summarizes the results of the included studies. Eight of the 11 included studies found that PA interventions were cost-effective regarding at least one health outcome [22, 24–30], whereas the other 3 studies reported a lack of cost-effectiveness [23, 31, 32].

**Table 3:** Summary of cost-effectiveness findings of the reviewed studies

Study	Population	Intervention	Cost-effectiveness	Conclusion
Baal et al. [29] (2016)	No MCI or dementia	Increase of physical activity level by 1 level (out of total 5 levels) vs. no increase	Life years:+	If prevention is targeted at the physically inactive by increasing physical activity level, savings in dementia-related costs outweigh the additional spending in life years gained
Wimo et al. [30] (2022)	No MCI or dementia	2-year multidomain program (nutritional counseling, multicomponent exercise, and cognitive training) vs. standard-of-care	QALY: +	The model provides support that programs like FINGER have the potential to be cost-effective in preventing dementia
Kato et al. [22] (2022)	No MCI or dementia	90-min weekly combined physical and cognitive session	QALY: +	A program targeting community-dwelling healthy adults aged 65 years old could be cost-effective
Davis et al. [26] (2013)	MCI	6-month resistance training or aerobic training vs. balance/tone classes (control)	Executive cognitive function:+	Resistance training and aerobic training result in healthcare cost saving and are more effective than balance/tone classes in older adults with MCI. Resistance training is a promising strategy to alter the trajectory of cognitive decline in seniors with MCI
Davis et al. [27] (2017)	MCI	6-month aerobic training + standard-of-care + education vs. standard-of-care + education	QALY:+	Aerobic training represents an attractive and potentially cost-effective strategy for older adults with mild subcortical vascular cognitive impairment
Eckert et al. [28] (2021)	MCI	12-week home-based exercise (walking, balance, strength training) vs. flexibility training	Physical performance and QALY:+	The home-based exercise intervention demonstrated high probability of cost-effectiveness in terms of improved physical performance in older adults with MCI following discharge from ward rehabilitation. The intervention had high probability of being cost-effective in terms of QALY when using a high willingness to pay threshold
Khan et al. [31] (2019)	Dementia	12-month aerobic and resistance exercise classes vs. standard-of-care	Cognitive outcomes and QALY:–	Exercise is not cost-effective in slowing cognitive impairment in people with mild to moderate dementia
Pitkälä et al. [24] (2013)	Dementia	12-month group-based exercise or home-based exercise vs. standard-of-care	Physical functioning and mobility: + (home-based exercise) - (group-based exercise)	An intensive and long-term exercise program administrated at patient’s home had beneficial effects on the physical functioning of patients with Alzheimer's disease without increasing the total costs of health and social services or causing any significant adverse effects
Sopina et al. [32] (2017)	Dementia	16-week aerobic exercises (on bicycle, cross trainer, and treadmill) vs. usual treatment	QALY:–	The exercise intervention is unlikely to be cost-effective within the commonly applied threshold values
Van Santen et al. [23] (2021)	Dementia	6-month exergaming (interacting cycling) + regular activity program (music listening, singing, arts and crafts, cooking, gymnastics, and outdoor walking) vs. regular activity program	QALY, physical activity function and mobility:–	Exergaming by participants with dementia in daycare center was nor cost-effective compared to care as usual for our primary outcome measures: QALYs, physical activity and mobility
D’Amico et al. [25] (2016)	Dementia	12-week daily walking program vs. standard-of-care	BPSD: + QALY:–	For individuals with dementia, exercise could potentially be a cost-effective intervention for outcomes measured by BPSD, but not when measured by QALYs

Studies were grouped by the population and ordered by the severity of dementia. “ + ” means cost-effective and “–” means not cost-effective. HRQOL indicates for Health-related quality of life

*MCI *mild cognitive impairment*, FINGER *The Finnish Geriatric Intervention Study to Prevent Cognitive Impairment and Disability*, BPSD* behavioral and psychological symptoms of dementia, *QALY* quality-adjusted life years

Three studies evaluated the PA interventions implemented in individuals with no existing MCI or dementia and found PA interventions were cost-effective in increasing life year and quality-adjusted life year in the long term. Baal et al. investigated the relationship between increasing PA levels, the incidence of dementia, mortality, and the use of health care and social care in individuals with no dementia [29]. In this study, a simulation model was used to project various scenarios with different assumptions on increasing PA by one level among the English population aged 40–65. Preventing dementia by increasing PA was projected to increase life expectancy and decrease overall spending on health and social care, even after adjusting for additional spending during the life years gained [29]. Kato et al. estimated the cost-effectiveness of the combined physical and cognitive program designed to prevent community-dwelling healthy adults aged 65 years old from developing dementia. This study used a simulation model and found that the combined physical and cognitive program was not only effective in increasing quality-adjusted life years but also cost-saving during the 10-year period [22]. Wimo et al. estimated the potential cost-effectiveness of the Finnish Geriatric Intervention Study to Prevent Cognitive Impairment and Disability (FINGER) program [30]. The FINGER program was the first randomized control trial to show a statistically significant beneficial effect on cognition of a multidomain lifestyle intervention program (including diet, PA, and cognitive training) for older adults at risk of developing dementia. Wimo et al. used a simulation model and projected that a multidomain lifestyle intervention program was cost-saving and clinically superior in preventing dementia than standard-of-care.

Three studies evaluated the PA interventions that implemented in individuals with MCI and found PA interventions were cost-effective in improving cognitive function, physical performance, and quality-adjusted life year in the short term. Davis et al. found that either a 6-month aerobic or resistance training was more cost-effective in improving executive cognitive function than other exercises focused on improving balance and muscle tone in older adults with MCI [26]. Davis et al. also found that a 6-month aerobic training PA program was more cost-effective in maintaining the health-related quality of life compared to standard-of-care in individuals with subcortical ischemic vascular cognitive impairment, a subtype of MCI [27]. Eckert et al. found that a 12-week home-based personalized PA program (that included exercises for balance and strength as well as walking) was more cost-effective than a flexibility training program in terms of improving physical performance and quality-adjusted life year [28].

In the 5 studies evaluated the PA interventions that implemented in individuals with dementia, we found mixed results regarding the cost-effectiveness of PA interventions. D’Amico et al. studied a dyadic PA program, which delivered PA in the form of a 12-week individually tailored walking program lasting for 20–30 min daily and designed to become progressively more intensive [25]. D’Amico et al. concluded that this dyadic PA program may be cost-effective for improving behavioral and psychological symptoms of dementia but not for improving quality-adjusted life year [25]. Pitkälä et al. found that a 12-month home-delivered, personalized PA program delayed the expected deterioration in physical function among individuals with dementia without increasing total health and service costs of standard-of-care [24]. However, in the same study, Pitkälä et al. also found that the paralleled 12-month group-based PA intervention with nondistinctive PA sessions was not cost-effective compared with standard-of-care [24]. Khan et al. found that a 12-month moderate-to-high-intensity aerobic and strength training PA program was not cost-effective compared with standard-of-care for individuals with mild to moderate dementia [31]. The results showed that this PA program did not significantly affect cognitive outcomes or quality-adjusted life years. Similarly, Sopina et al. found that a 16-week moderate-to-high intensity aerobic training PA program was not cost-effective in terms of participant-reported and proxy-reported health-related quality of life [32]. Van Santen et al. also studied the cost-effectiveness of “exergaming,” an innovative form of PA that integrates physical activity with cognitive stimulation in a gaming environment, and found that exergaming was not cost-effective compared to standard-of-care in improving quality-adjusted life year, physical function, and mobility for individuals with dementia [23].

Two studies evaluated a 12-week PA intervention but had different outcomes. One of the studies targeted individuals with MCI, while the other targeted individuals with dementia. Eckert et al. found that a 12-week home-based tailored exercise program was cost-effective in improving physical performance and quality-adjusted life year among individuals with MCI at a relatively low willingness-to-pay threshold (i.e., equate to £18,000/quality-adjusted life year) [28]. In comparison, delivery of a PA program with the same duration of time was found to be less cost-effective among individuals with dementia. D’Amico et al. found that walking at least 20 min daily for 12 weeks for individuals with dementia was cost-effective when considering behavioral and psychological symptoms of dementia. However, it was not cost-effective in terms of quality-adjusted life year, even considering a high willingness-to-pay threshold (i.e., £30,000/quality-adjusted life year) [25].

## Discussion

### Main findings

This review provides an expanded discussion on the effectiveness of PA interventions [9–11] by evaluating the economic impact and cost-effectiveness of these interventions as they relate to populations with or without existing MCI or dementia. Our results indicate that an intensive PA program implemented before MCI diagnosis may be cost-effective in the long term. Also, PA programs may be cost-effective in the short term among individuals with MCI. However, we did not find sufficient evidence on the long-term cost-effectiveness of PA interventions in individuals with MCI or existing dementia.

In evaluating evidence supporting PA intervention as a prevention strategy in individuals with no existing dementia diagnosis, it is important to note that two studies may provide an overly conservative estimate of the health benefit of the PA interventions in individuals with MCI, given that participants may also have experienced a positive health benefit from the control interventions that included PA [26, 28]. Therefore, the evidence on the short-term cost-effectiveness of prevention strategies for dementia among individuals with MCI is likely underestimated. Our review found that even a short PA program (i.e., 12-week PA intervention) showed cost-effectiveness for individuals with MCI. To be cost-effective, PA interventions may need to be implemented earlier in life before dementia diagnosis. Short-term PA interventions that begin after the onset of dementia are likely insufficient to provide beneficial effects when considering economic sustainability. Increasing PA levels among physically inactive adults was projected to be cost-saving as a prevention strategy for dementia over the simulated lifetime, whereas the cost of PA intervention was not considered in the study [29]. Also, the dominance (i.e., both clinically superior and cost-saving) demonstrated in the two multidomain programs (i.e., the combined physical and cognitive program and the multidomain lifestyle intervention program) that addressed several dementia risk factors was intriguing [22, 30]. Although it was not possible to isolate the benefits attributable to the PA domain, given the complex relationship between interrelated factors that protect against cognitive decline and dementia, interventions addressing many risk factors at once might offer the best prevention strategy for individuals who already at higher risk for cognitive impairment or dementia.

In evaluating evidence on whether PA interventions are cost-effective in patients with current dementia, we found mixed results. The lack of cost-effectiveness documented in some studies may have been due to limited clinical benefits of the PA intervention, lack of compliance with the intervention among individuals with dementia, or the intervention being too costly. For example, it is possible that the clinical benefits in physical function and mobility did not translate into improvements in functional activities that may have been required to demonstrate improved cognitive outcomes. The two studies reporting the cost-effectiveness of PA interventions in current dementia patients showed the importance of participant compliance and intervention cost in determining outcomes. One study evaluated a 12-week walking program, and the other evaluated a 12-month home-based exercise program [24, 25]. Even with limited clinical benefits, the low financial investment of implementing a walking program may substantially contribute to the cost-effectiveness of the intervention [25]. Additionally, adherence was reported to be exceptionally high in the 12-month home-based PA program with personalized training, ensuring high levels of activity that lasted for an intense and sufficient duration of PA [24]. Thus, it is reasonable to expect that low cost is conducive to high compliance, which leads to higher benefits the participant reaps from the intervention. Although it was not possible to extrapolate these findings to other programs due to the substantial heterogeneity across studies and PA programs, it is possible that other 12-month PA programs (e.g., the 12-month PA program with aerobic and resistance exercise classes reviewed in this study [31]) would have been more cost-effective if a higher adherence was achieved.

### Current gaps and future directions

Among the reviewed studies, only the model-based and “hybrid” CEA assess the long-term cost-effectiveness of PA interventions. In contrast, other studies used trial-based data to evaluate short-term cost-effectiveness. Clinical trials are often limited by finite (and potentially short) follow-up duration, and pure trial-based CEA may not have sufficient data to report long-term (e.g., lifetime) costs and the consequences of PA interventions [18]. Previous research demonstrates that PA, such as resistance training in older adults, has long-term health benefits and economic impact that a longer time horizon would ideally capture [26, 35]. Therefore, it is possible that the time horizons of the reviewed trial-based CEA were not sufficiently long to capture all the pertinent clinical and economic ramifications of the strategies under study, and the estimation of cost-effectiveness may be biased [18]. Moreover, pure trial-based analyses tend not to incorporate data from external sources, exposing the results to potentially greater uncertainty than if evidence from other prospective studies or trials was considered. Further investigation on the long-term cost-effectiveness of PA intervention, both as a prevention strategy and a management strategy for dementia, is warranted. When clinical trials looking at long-term costs and consequences are not feasible or complete, simulation models can be applied to estimate likely cost-effectiveness outcomes by incorporating data from a wide variety of sources as inputs. In particular, “hybrid” studies can address the limitations of trial-based CEA—the issue of truncated follow-up—by extending the results of the study through time, generating a range of plausible projections of longer-term outcomes [18, 30]. Moreover, as most of the identified studies were confined to a European setting, CEA conducted in other settings is warranted.

### Limitations

The interpretation of this systematic review may be limited by the nature of narrative synthesis. Additionally, a meta-analysis was not feasible due to the substantial heterogeneity among the small number of identified studies. First, there were multiple sources of heterogeneity, such as population variations and measurement of CEA outcomes. Second, the studies were conducted from different perspectives in different settings with various willingness-to-pay thresholds. Third, there is structural and methodological heterogeneity between model-based and trial-based CEA. While the sample size is often used to weigh the impact of each study included in a meta-analysis, it needs to be clarified how to assign weights to model-based studies [36].

## Conclusions

Our review identifies population traits and intervention characteristics that trend toward the cost-effectiveness of PA interventions to prevent and manage cognitive decline and dementia. PA interventions administrated in middle-aged or older individuals prior to MCI or dementia diagnosis were generally cost-effective in the long term. We also found short-term cost-effectiveness of PA interventions among individuals with MCI, who may represent a target population in urgent need of cost-effective lifestyle-modified interventions, given that they have not yet crossed the dementia threshold.

Future studies should further explore the long-term cost-effectiveness of PA interventions among individuals with MCI, who are at increased risk for eventual dementia diagnosis. Also, more CEA should be conducted in settings other than Europe. Although we found mixed results on the cost-effectiveness of PA interventions for individuals with existing dementia, there may still be a benefit of implementing PA strategies in this population because it could also provide cognitive benefits, as has been shown for other chronic conditions (e.g., cardiovascular diseases) [37, 38]. A lower cost burden and implementation strategies to improve adherence might be key factors in achieving the cost-effectiveness of PA interventions in individuals with dementia.

### Supplementary Information

**Additional file 1.** PRISMA 2020 Checklist.

**Additional file 2:** **Table S1.** Comprehensive search strategy for the systematic review - databases and keywords used.

## Data Availability

Data sharing is not applicable to this article as no datasets were generated or analyzed during the current study.
