# Supplementary material for: Cost-effectiveness of physical activity interventions for prevention and management of cognitive decline and dementia—a systematic review
Source: Alzheimers Res Ther. 2023 Sep 25;15:159. doi: 10.1186/s13195-023-01286-7 (PMC10519096; doi:10.1186/s13195-023-01286-7)
Supplement: Supplementary file 2 — Additional file 2: Table S1. Comprehensive search strategy for the systematic review - databases and keywords used. [file 13195_2023_1286_MOESM2_ESM.docx]

Table S1. Comprehensive search strategy for the systematic review - databases and keywords used.

| Database | Search terms or keywords |
| --- | --- |
| PubMed | ((physical exercise) OR (physical activity) OR (leisure time) OR (sport) OR (muscle stretching exercise) OR (fitness) OR (physical activities) OR (exercise training) OR (physical training)) AND ((quality-adjusted) OR (cost-utility) OR (cost-effectiveness) OR (health economics) OR (economic evaluation)) AND ((mild cognitive impairment) OR (dementia) OR (alzheimer's disease)) |
| Embase | ('exercise' OR 'physical activity' OR 'leisure' OR 'sport' OR 'stretching exercise' OR 'fitness' OR 'training') AND ('quality adjusted life year' OR 'cost effectiveness analysis' OR 'cost utility analysis' OR 'health economics' OR 'economic evaluation') AND ('mild cognitive impairment' OR 'dementia' OR 'alzheimer disease') |
| Science Direct | ('exercise' OR 'leisure time ' OR 'sport' OR 'fitness') AND ('cost effectiveness analysis' OR 'cost utility analysis') AND ('mild cognitive impairment' OR 'dementia' OR 'alzheimer disease') |
